# Predictors of COVID-19 vaccine uptake among adults in South Africa: multimethod evidence from a population-based longitudinal study

**DOI:** 10.1136/bmjgh-2023-012433

**Published:** 2023-08-04

**Authors:** Brendan Maughan-Brown, Katherine Claire Eyal, Lindokuhle Njozela, Alison M Buttenheim

**Affiliations:** 1 Southern Africa Labour and Development Research Unit, University of Cape Town, Cape Town, South Africa; 2 Southern Africa Labour and Development Research Unit, School of Economics, University of Cape Town, Cape Town, South Africa; 3 School of Economics, University of Cape Town, Cape Town, South Africa; 4 Family and Community Health, University of Pennsylvania School of Nursing, Philadelphia, Pennsylvania, USA

**Keywords:** Vaccines, COVID-19, Public Health, Cohort study

## Abstract

**Background:**

COVID-19 vaccine coverage remains low in many low and middle-income countries despite widespread access. To understand the dynamic decision-making process around vaccination and provide evidence for future vaccine promotion campaigns, we identified predictors of COVID-19 vaccine uptake among South African adults, including those who did not believe in the vaccine’s safety or efficacy.

**Methods:**

Data from two longitudinal telephone surveys in late 2021 and early 2022 of unvaccinated South African adults were used to model COVID-19 uptake. Predictors of interest informed by the theory of planned behaviour included vaccine attitudes and beliefs, social norms, perceived behavioural control and vaccine intentions. Responses to open-ended questions provided insights into key reasons for getting vaccinated.

**Results:**

Among panel participants (n=1772), 19% reported being vaccinated between Survey 1 and Survey 2. Vaccine uptake was greater among participants who reported wanting to get vaccinated ‘as soon as possible’ (+27 percentage points, p<0.01). Vaccine uptake was greater among participants who believed that the vaccine is effective in preventing COVID-19 infection and/or death (+12 percentage points, p<0.01) and lower among those who believed that the vaccine is unsafe (−9 percentage points, p<0.01). Among participants who did not believe the vaccine is safe, living with someone already vaccinated against COVID-19 increased vaccine uptake (+6 percentage points, p<0.05). At Survey 1, the intention to get vaccinated as soon as possible was positively associated with perceived risk of illness from COVID-19 (+9.2 percentage points, p<0.05), the belief that most people in their area had been vaccinated (+7.0 percentage points, p<0.05) and living with someone already vaccinated against COVID-19 (+6.6 percentage points, p<0.05).

**Conclusion:**

Study findings underscore the predictive power of intentions and of beliefs about disease risk, vaccine safety and vaccine efficacy. Social proof interventions hold promise for increasing vaccination intentions and overcoming concerns about vaccine safety.

**WHAT IS ALREADY KNOWN ON THIS TOPIC**

- A growing body of evidence has documented correlates of COVID-19 vaccination intentions and hesitancy, but there is limited evidence globally on the predictors of actual COVID-19 vaccine uptake.

**WHAT THIS STUDY ADDS**

- Perceiving COVID-19 vaccination as the social norm and living with someone vaccinated increased vaccination intentions and uptake.
- Subgroup analyses found that perceived risk from COVID-19 and perceived vaccine efficacy influenced vaccine uptake among individuals concerned about vaccine safety; and perceived personal risk from COVID-19 predicted vaccine uptake among individuals who did not think that the vaccine works.

**HOW THIS STUDY MIGHT AFFECT RESEARCH, PRACTICE OR POLICY**

- Results indicate that some individuals who do not believe the vaccine works will get vaccinated anyway if they perceive themselves at risk of illness; and that for some the perceived risks from getting vaccinated can be outweighed by the personal health benefits from vaccination.
- Our findings highlight the potential of social proof interventions as one way to overcome concerns about vaccine safety as a barrier to vaccination.

## Introduction

The COVID-19 pandemic has caused at least 7.0 million deaths worldwide, and likely many more given excess mortality estimates.^1 2^ Since their widespread availability in early 2021, COVID-19 vaccines have been central to curbing the spread and reducing the burden of the disease.^3^

However, despite rapid, widespread access to COVID-19 vaccines, vaccination coverage remains low in many low and middle-income countries.^4^ In South Africa, only 25% of the adult population was vaccinated in the first 6 months of general population eligibility (May to December 2021).^4^ While vaccine uptake was initially restricted by poor access,^5^ increased vaccine access and extensive national demand-creation activities^6^ failed to improve vaccine coverage significantly. With future waves of COVID-19 infection expected, and with waning efficacy of initial vaccines, it remains imperative to increase COVID-19 vaccine coverage in South Africa and globally.^7^

The unprecedented pace of COVID-19 vaccine development contributed to widespread concerns over vaccine efficacy and safety.^8–10^ A growing body of evidence points to greater reluctance to get vaccinated among those concerned about the safety of COVID-19 vaccines^5 11–13^ and among those who do not believe they are effective.^14 15^

In contrast, there is limited evidence globally on the predictors of actual COVID-19 vaccine uptake, a more challenging outcome to measure. One study in Hong Kong found that intention to get vaccinated did not predict future vaccination.^16^ This underscores both the fact that intentions do not always translate into behaviours^17^ and the importance of evidence on predictors of vaccine uptake for the design of vaccine demand generation campaigns. The Hong Kong study demonstrated that COVID-19 vaccine uptake was more likely among adults who knew others who had been vaccinated against COVID-19, who trusted authorities more, who found non-pharmaceutical prevention measures acceptable and who had first-hand experience of COVID-19.^16^ A recent study in South Africa, using data from the same survey as our study, found that among black African participants the factors most influential in decisions on COVID-19 vaccination were low perceived risk and concerns about vaccine efficacy and vaccine safety.^18^

To understand COVID-19 vaccine uptake we conducted a longitudinal telephone survey in South Africa several months after the start of the COVID-19 vaccination roll-out. Our central aim was to identify the factors that predicted COVID-19 vaccine uptake among the adult population. Given previous studies demonstrating the predictive power of beliefs about vaccine safety and efficacy for vaccination intentions,^18^ an important secondary study aim was to identify factors that predicted COVID-19 vaccine uptake among adults holding such beliefs. As most studies of COVID-19 vaccine intentions and behaviour have been cross-sectional, our study adds to the limited evidence on the dynamic decision-making process of those who chose not to get vaccinated when the vaccine was first available. We use the theory of planned behaviour (TPB)^19–21^ to examine the influence of attitudes/beliefs, subjective norms and perceived behavioural control on both vaccine intentions and uptake. Our analyses aimed to answer four questions: (1) Did attitudes/beliefs, norms and behavioural control predict COVID-19 vaccine intentions? (2) Did intentions to get vaccinated predict vaccine uptake? (3) Did attitudes, norms and perceived behavioural control predict vaccine uptake? (4) Did vaccine intentions mediate the effect between attitudes/beliefs, norms and perceived behavioural control and vaccine uptake? Responses to open-ended questions provide qualitative insights on respondents’ main reasons for getting vaccinated. Findings offer insights relevant for efforts to increase early adoption of a vaccine, as well as to increase demand among those initially reluctant to get vaccinated.

## Methods

### Study setting and design

**Figure 1:**
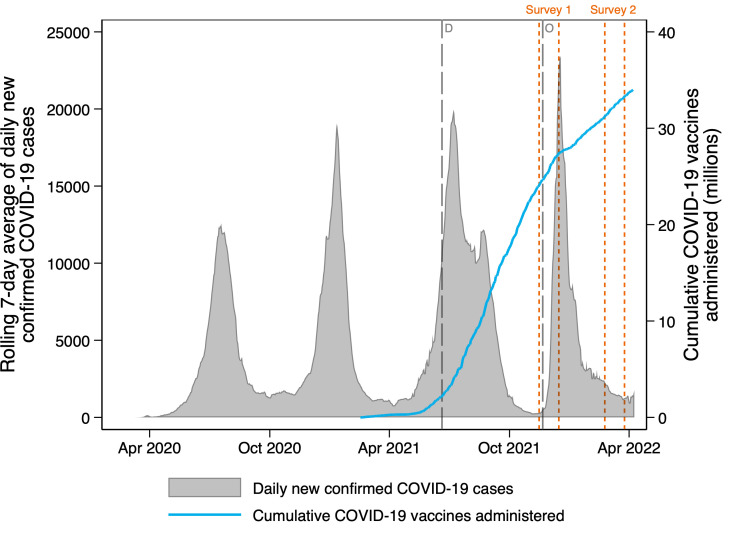
Timing of the COVID-19 Vaccine Survey (CVACS) in the context of COVID-19 infections and vaccinations administered in South Africa. ‘D’ indicates when the president of South Africa announced the Delta variant. ‘O’ indicates when the president of South Africa announced the Omicron variant.

A longitudinal study—The COVID-19 Vaccine Survey (CVACS)—was conducted among the general population in South Africa. CVACS collected information on facilitators and barriers to COVID-19 vaccine uptake to support vaccine demand-creation strategies. Two telephone surveys were conducted (see figure 1).

Survey 1 (15 November to 15 December 2021) was conducted about 9 months after the first vaccines were administered in South Africa in February 2021 to healthcare workers participating in the Sisonke Phase 3b Implementation trial of the single-dose Janssen COVID-19 vaccine, Johnson & Johnson.^22^ The two-dose Pfizer–BioNTech COVID-19 vaccine was used in the phased (age based) general population programme: ≥60 years in May, ≥35 years in July, ≥18 years in August 2021. Only about 25% of the adult population was vaccinated during May to December 2021.^4^ In this study, the term ‘the vaccine’ is used to refer to COVID-19 vaccines in general, rather than to either specific vaccine, but most of the study participants would have received the Pfizer vaccine. CVACS Survey 1 was conducted during the rise of the fourth wave of COVID-19 infections, with 93% of interviews conducted after the South African Presidential Address (28 November) announcing the identification of the Omicron variant. Survey 2 (23 February to 25 March 2022) was administered approximately 3 months later, which coincided with the end of the fourth wave of COVID-19-related deaths (January to March 2022).^23^ Survey 2 attempted to reinterview the baseline participants, with a lucky draw implemented to increase retention in the panel. CVACS Survey 1 data^24^ and Survey 2 data^25^ are publicly available.

### Study population and data collection

Individuals aged 18 and older living in South Africa who self-reported not being vaccinated against COVID-19 were eligible to participate in the study. Data were collected through computer-assisted telephone interviewing, with interviews conducted in 11 official languages. The Survey 1 sample was drawn from a large credit bureau database, which includes individuals who had applied for credit, regardless of the outcome, and individuals who have had a credit check. The sample frame consisted of over 16 million contact numbers^26^ and comprised individuals across the socioeconomic spectrum. The sample was stratified on several characteristics to ensure representation across provinces, urbanicity, age groups (based on the age categories used for the national vaccine roll-out), gender and income groups.^26^

### Patient and public involvement

The CVACS survey instrument design process involved the crowdsourcing of policy-relevant questions from the public.

### Measures

#### Outcome variables

*COVID-19 vaccine intention at Survey 1* was measured by asking participants ‘Regarding the COVID-19 vaccine, do you plan to: 1. Get it as soon as possible, 2. Wait and see, 3. Only get it if it is required (for example, if it is required for school or work) or 4. Definitely not get it?’ A binary variable was created to identify individuals who reported they wanted to get vaccinated as soon as possible (=1) from the rest of the sample (=0).

*COVID-19 vaccine uptake at Survey 2*: Participants were asked ‘Have you received at least one injection of a Coronavirus vaccine?’, with dichotomous ‘yes’/‘no’ responses captured.

*Reasons for getting vaccinated*: Participants who reported being vaccinated in Survey 2 were asked two open-ended questions: (1) ‘What is the main reason you decided to get vaccinated?’; (2) ‘Last time we spoke to you, you were not yet vaccinated. Since then, what is the main thing that changed or helped you decide to get vaccinated?’

#### Independent (predictor) variables (baseline: Survey 1)

Our analysis of factors predicting COVID-19 vaccine uptake was informed by the theory of planned behaviour (TPB), which has been used to explain many health-related behaviours.^27–30^ In relation to COVID-19 vaccine uptake, TPB posits that vaccine uptake is determined by intentions to get vaccinated, which are influenced by attitudes and beliefs (about both COVID-19 and COVID-19 vaccines), social norms (perceptions and knowledge of whether others are getting vaccinated) and perceived behavioural control (perception of the difficulty of getting vaccinated).

In our study, *perceived personal COVID-19 risk* was measured using the question ‘Do you think you will get very sick with COVID-19 in the next 12 months?’ (yes/no). Perceived efficacy of the COVID-19 vaccine was assessed using two measures: (1) ‘Do you believe that the COVID-19 vaccine would help to prevent you from getting COVID-19?’ (yes/no); (2) ‘Do you believe that the COVID-19 vaccine will help to prevent you from dying from COVID-19?’ (yes/no). A binary variable (*COVID-19 vaccine is effective*) was created where 1=believes that the vaccine prevents COVID-19 infection and/or prevents death and 0=believes neither of those things. Concerns about the safety of COVID-19 vaccines were measured by asking participants to respond ‘yes’/‘no’ to the following reasons for why they had not yet vaccinated: believes the vaccine may kill you and does not trust the safety of the COVID-19 vaccine. A binary variable (*COVID-19 vaccine is unsafe*) was created to indicate individuals who reported that they did not trust the safety of the COVID-19 vaccine and/or believed that the vaccine may kill them.

Social norms relating to COVID-19 vaccination (*perceived vaccination coverage*) were measured with the question ‘Do you think most, some, or hardly any people living in your area have been vaccinated for COVID-19?’ In addition, a measure of close contact with someone who had been vaccinated against COVID-19 (*lives with someone vaccinated*) was collected by asking whether any of the people living with the participant had been vaccinated.

Perceived behavioural control of getting vaccinated against COVID-19 was assessed by summing ‘yes’ responses to four potential access-related barriers as reasons for not yet having been vaccinated: *do not have time to go to get vaccinated; do not know where to go to get vaccinated; vaccination site is too far away*; and *travel costs to the vaccination site are too high*. A scale (0–4) for *perceived vaccine access barriers* was created by summing these four binary measures.

#### Covariates (baseline: Survey 1)

Demographic measures were age (continuous), gender, race, province of residence and geotype of residence (traditional area/chiefdom, urban area/town, farm/rural area). Socioeconomic variables included education (highest grade completed), household hunger (past 7 days), vehicle ownership and total household income (last month). Experience of COVID-19, which could potentially influence attitudes and beliefs, was captured with two measures: (1) ‘Have you had COVID-19?’ (yes/no); (2) ‘Do you know anyone personally who has died from COVID-19 or gotten very sick with COVID-19?’ (yes/no).

### Statistical analysis

We report proportions or mean scores of demographic, socioeconomic status and COVID-19-related variables and assess significant differences in those measures by our primary outcome variables (vaccination intention in Survey 1 and vaccine uptake by Survey 2) using χ^2^ tests (differences in proportions) and analysis of variance for differences in mean scores.

#### Factors associated with COVID-19 vaccination intentions

Multivariable ordinary least squares (OLS) regression was used to assess correlates of COVID-19 vaccine intentions at Survey 1. OLS was selected due to ease of interpretation. The binary dependent variable (measured at Survey 1) distinguished individuals who reported wanting to get vaccinated as soon as possible (=1, ie, high intent) from the rest of the sample (=0, ie, lower intent). Independent variables relating to the TPB (measured at Survey 1) included measures of attitudes and beliefs, social norms and perceived behavioural control described above. All models controlled for potential covariates and applied Survey 1 design weights. For consistency with analyses of predictors of vaccine uptake (below), the sample for this analysis was restricted to the balanced panel (ie, individuals who were interviewed in both Survey 1 and Survey 2). Sensitivity analyses were conducted using the full Survey 1 sample.

#### Predictors of COVID-19 vaccine uptake

Predictors of vaccine uptake (by Survey 2) were assessed among the balanced panel using multivariable OLS regression with panel weights applied. To assess whether intentions predicted vaccine uptake, we included vaccine intentions as the only independent variable of interest in the model. To assess whether attitudes/beliefs, social norms and perceived behavioural control (independent of intentions) influenced vaccine uptake, these measures were included in a separate model, with the intention measure excluded. We then included all independent variables of interest in the model to assess whether vaccine intentions mediated the effect between attitudes/beliefs, social norms and perceived behavioural control and vaccine uptake.

Subgroup analyses were then conducted by replicating the models of potential predictors of COVID-19 vaccination uptake among two groups with perceptions or beliefs that have been shown to act as barriers to COVID-19 vaccine intentions: (1) those not trusting the safety of the vaccine^5 11 12 31–33^ and (2) those not believing the vaccine to be effective.^14 34^ The subgroup analyses aimed to identify factors that could potentially be leveraged to encourage vaccine uptake among individuals with common concerns about the vaccine.

In all analyses, p values <0.05 were considered statistically significant. Sensitivity analyses replicated the linear regression models using logistic regression to assess whether results were substantively similar across different models. Analyses were conducted in Stata V.17.^35^

### Main reasons for getting vaccinated: Survey 2 qualitative responses

Open-ended responses on reasons for getting vaccinated were analysed using a content analysis approach.^36 37^ A codebook was developed by two researchers who independently analysed 200 responses from each question and categorised responses based on the emerging themes, the literature (ie, themes analysed or found in other studies) and applicable behavioural theory. Following common practice,^38^ the full set of responses was then double coded by independent coders using the codebook, with differences reconciled using a separate third coder blinded to the contradictory codes. Remaining inconsistencies between the three coders were reconciled by the study team, with unclear or ambiguous responses classified as uncategorised.

As our study team involved an international partnership, an author reflexivity statement was developed using guidance on promoting equitable authorship in publications^39 40^ and can be found in online supplemental appendix S1.

10.1136/bmjgh-2023-012433.supp2 Supplementary data

## Results

### Participant inclusion

Among 14 577 successfully contacted and screened for study eligibility, 10 536 individuals (72%) reported being vaccinated, 3969 (27%) reported being unvaccinated, 64 refused and 8 reported ‘don’t know’. The final Survey 1 sample of individuals unvaccinated against COVID-19 was 3510, after 459 unvaccinated were excluded for a range of reasons including partially completed interviews. In Survey 2, 1772 of the initial Survey 1 participants were reinterviewed (attrition rate of 50%). Not being reachable was the primary reason for survey attrition. This may, in part, be due to participants changing cellphone numbers, which is common practice in South Africa.^41^

### Participant characteristics

The unweighted panel sample (interviewed in both Survey 1 and Survey 2) was 53% men, had an average age of 40 and had almost 11 grades of completed education (table 1). Approximately 58% lived in households earning less than R5000 per month (equivalent to US$315 midpoint of Survey 1) and nearly one in five households did not have enough to eat in the past 7 days. One-third intended to get vaccinated against COVID-19 ‘as soon as possible’. Perceived risk of COVID-19-related illness was low (13%), fewer than half (43%) believed the vaccine was effective in preventing infection/death and concerns about vaccine safety were common (67%). One in three believed COVID-19 vaccination was the social norm in their area, and 42% lived with someone already vaccinated. The proportion reporting each access-related barrier to vaccination ranged from 16% to 28%.

**Table 1:** Sample characteristics and beliefs for the full sample and by vaccination status (with tests of significance of the difference between vaccinated and unvaccinated participants)

	Overall %/mean (n) unweighted	Overall %/mean weighted	Unvaccinated %/mean weighted	Vaccinated %/mean weighted	P value difference
Male	53.45% (1772)	54.25%	55.80%	47.79%	0.052
Age (years, mean)	40.16 (1771)	36.73	36.87	36.16	0.363
Grade completed (mean)	10.73 (1766)	10.99	11.02	10.83	0.185
Household income >R5000	41.68% (1336)	41.19%	42.38%	36.19%	0.179
Household went hungry (past 7 days)	18.14% (1747)	19.94%	19.77%	20.60%	0.796
Vaccine intention: As soon as possible	34.24% (1767)	31.91%	24.60%	62.57%	<0.01
Perceived personal COVID-19 risk	13.1% (1770)	12.44%	10.98%	18.53%	0.015
Believes COVID-19 vaccine is effective	42.89% (1770)	41.23%	35.56%	64.88%	<0.01
Believes COVID-19 vaccine is unsafe	67% (1769)	69.29%	73.50%	51.77%	<0.01
Perceived vaccination coverage: ‘Most’ people	35.4% (1770)	36.10%	33.86%	45.50%	<0.01
Lives with someone vaccinated	41.77% (1772)	43.61%	42.00%	50.35%	0.041
Reason not vaccinated: Does not have time	28.18% (1767)	25.70%	23.51%	34.83%	<0.01
Reason not vaccinated: Do not know where to go	15.54% (1767)	12.44%	11.07%	18.14%	0.012
Reason not vaccinated: Site too far away	16.79% (1768)	14.95%	13.49%	21.02%	0.014
Reason not vaccinated: Transport too costly	17.93% (1765)	17.28%	16.49%	20.57%	0.184
Has had COVID-19	15.61% (1769)	13.06%	12.70%	14.59%	0.51
Knows someone who has had COVID-19	48.32% (1769)	49.09%	49.60%	46.99%	0.522

Data are weighted using panel weights.

Differences in weighted proportions showed that those vaccinated against COVID-19 (by Survey 2) had reported (in Survey 1) greater intentions to get vaccinated, and were more likely to have perceived themselves at risk of illness, to have believed the vaccine is effective and vaccination is the social norm. Unvaccinated participants reported greater concern about the safety of vaccines. Counterintuitively, vaccinated participants reported more access-related barriers.

### Correlates of COVID-19 vaccination intentions

All three measures of attitudes and beliefs were significantly associated with vaccine intention (ie, plan to get vaccinated as soon as possible, see table 2, model 1). Participants were more likely to intend to get vaccinated if they thought they might get sick with COVID-19 within 12 months (+9.2 percentage points, p<0.05), and if they believed that the vaccine is effective in preventing COVID-19 infection and/or death (+31.6 percentage points, p<0.01). Participants who believed that the vaccine is unsafe were less likely to intend to get vaccinated (−19.6 percentage points, p<0.01). Stated vaccine intentions were also associated with social norms, with intentions greater among participants who believed that most people in their area had been vaccinated (+7.0 percentage points, p<0.05, compared with those who believed hardly anyone or did not know), and among participants who lived with someone already vaccinated against COVID-19 (+6.6 percentage points, p<0.05). The association between perceived behavioural control and vaccine intention ran in the opposite direction to TPB predictions: those with greater access-related barriers were more likely to plan to get vaccinated as soon as possible (+8.0 percentage points per scale point, p<0.01). Model 2 (table 2) replaced the access barrier scale with each binary component of the scale to explore this further. All components had a positive association, with not having time strongly associated with plans to get vaccinated as soon as possible (+12.2 percentage points, p<0.01).

**Table 2:** Multivariable ordinary least squares regression models of COVID-19 vaccine intention (dependent variable: plans to get vaccinated as soon as possible, Survey 1) with control variables

	Model 1 β (95% CI)	Model 2 β (95% CI)
Perceived personal COVID-19 risk	0.092** (0.004–0.180)	0.091** (0.004–0.178)
Believes COVID-19 vaccine is effective	0.316*** (0.253–0.380)	0.313*** (0.250–0.377)
Believes COVID-19 vaccine is unsafe	−0.196*** (−0.257 to −0.135)	−0.192*** (−0.253 to −0.132)
Perceived vaccination coverage: ‘Some’ people (Ref: Hardly any/Don’t know)	−0.003 (−0.061 to 0.055)	−0.004 (−0.061 to 0.054)
Perceived vaccination coverage: ‘Most’ people (Ref: Hardly any/Don’t know)	0.070** (0.004–0.136)	0.069** (0.004–0.135)
Lives with someone vaccinated	0.066** (0.014–0.117)	0.065** (0.014–0.116)
Access barriers (scale 0–4)	0.080*** (0.053–0.108)	
Reason not vaccinated: Does not have time		0.122*** (0.058–0.187)
Reason not vaccinated: Do not know where to go		0.057 (−0.022 to 0.135)
Reason not vaccinated: Site too far away		0.060 (−0.028 to 0.148)
Reason not vaccinated: Transport too costly		0.074* (−0.003 to 0.151)
Control variables included	Yes	Yes
Observations	1707	1707
R^2^	0.363	0.365

***P<0.01, **p<0.05, *p<0.1.

Results were substantively similar in unadjusted (online supplemental table S1), logistic (online supplemental table S2) and full Survey 1 sample (online supplemental table S3) models.

10.1136/bmjgh-2023-012433.supp1 Supplementary data

### COVID-19 vaccine uptake

Three hundred and eighty-six CVACS participants self-reported having received at least one dose of a coronavirus vaccine at Survey 2, a vaccination rate of 19% between November/December 2021 and March 2022. The equivalent vaccination rate among all adult South Africans unvaccinated by mid-December 2021 was approximately 7.5% over the same period.^4^ The higher vaccination rate within the study sample was likely driven by selection bias arising from survey attrition and sample selection (ie, those who agreed to be reinterviewed may have been more likely to have been vaccinated).

### Predictors of COVID-19 vaccine uptake

Vaccine intention was strongly associated with vaccine uptake (table 3, models 1 and 2). Participants who reported in Survey 1 that they wanted to get vaccinated as soon as possible were 27.2 percentage points more likely (p<0.01) to get vaccinated than other participants (model 1). Using the categorical measure of intentions, with the reference group being those who reported ‘wait and see’ in Survey 1, individuals who wanted to get vaccinated as soon as possible were 24.4 percentage points more likely (p<0.01) to get vaccinated, and those who definitely did not want to get vaccinated were 7.8 percentage points less likely (p<0.01) to get vaccinated (model 2).

**Table 3:** Multivariable ordinary least squares regression models of COVID-19 vaccine uptake (dependent variable: vaccine status in Survey 2)

	Model 1 β (95% CI)	Model 2 β (95% CI)	Model 3 β (95% CI)	Model 4 β (95% CI)	Model 5 β (95% CI)
Vaccine intention: As soon as possible (Ref: Everyone else)	0.272*** (0.209–0.335)			0.212*** (0.134–0.290)	
Vaccine intention: Definitely not (Ref: ‘wait and see’)		−0.078***(−0.129 to −0.027)			−0.055 (−0.108 to −0.003)
Vaccine intention: Only if required (Ref: ‘wait and see’)		−0.021 (−0.081 to 0.038)			−0.007 (−0.066 to 0.052)
Vaccine intention: As soon as possible (Ref: ‘wait and see’)		0.244*** (0.172–0.315)			0.201*** (0.119–0.284)
Vaccine intention: Don’t know (Ref: ‘wait and see’)		0.018 (−0.102 to 0.139)			0.025 (−0.095 to 0.145)
Perceived personal COVID-19 risk			0.079* (−0.013 to 0.172)	0.059 (−0.027 to 0.146)	0.060 (−0.026 to 0.146)
Believes COVID-19 vaccine is effective			0.124*** (0.064–0.183)	0.057* (−0.005 to 0.119)	0.049 (−0.014 to 0.112)
Believes COVID-19 vaccine is unsafe			−0.090*** (−0.151 to −0.030)	−0.049 (−0.110 to 0.013)	−0.048 (−0.109 to 0.014)
Perceived vaccination coverage: ‘Some’ people (Ref: Hardly any/Don’t know)			0.014 (−0.045 to 0.072)	0.014 (−0.042 to 0.070)	0.014 (−0.042 to 0.070)
Perceived vaccination coverage: ‘Most’ people (Ref: Hardly any/Don’t know)			0.050 (−0.015 to 0.115)	0.034 (−0.030 to 0.097)	0.032 (−0.031 to 0.095)
Lives with someone vaccinated			0.017 (−0.035 to 0.069)	0.004 (−0.045 to 0.053)	0.002 (−0.047 to 0.051)
Access barriers (scale 0–4)			0.022 (−0.005 to 0.049)	0.005 (−0.023 to 0.033)	0.003 (−0.024 to 0.031)
Control variables included	Yes	Yes	Yes	Yes	Yes
Observations	1717	1717	1709	1707	1707
R^2^	0.13	0.13	0.10	0.14	0.15

***P<0.01, **p<0.05, *p<0.1.

With vaccine intentions excluded from the analysis, two of the attitude/belief measures were significantly associated with vaccine uptake (table 3, model 3). Participants were more likely to get vaccinated if they believed that the vaccine is effective in preventing COVID-19 infection and/or death (+12.4 percentage points, p<0.01). Participants were less likely to get vaccinated if they believed that the vaccine is unsafe (−9.0 percentage points, p<0.01). Neither the subjective norms nor the behavioural control measures were significant predictors of vaccine uptake among the full balanced panel sample.

As expected (based on the TPB), in the full model with vaccine intentions included (table 3, models 4 and 5), only vaccine intentions remained a significant predictor of vaccine uptake.

Results were substantively similar in unadjusted (online supplemental table S4) and logistic (online supplemental table S5) models.

### Subgroup analysis: predictors of COVID-19 vaccine uptake among participants concerned about vaccine safety and among participants concerned about vaccine efficacy

Among participants who did not believe the vaccine is safe (table 4, model 1), participants were more likely to get vaccinated if they thought they may get sick with COVID-19 within 12 months (+12.9 percentage points, p<0.05), if they believed that the vaccine is effective in preventing COVID-19 infection and/or death (+8.7 percentage points, p<0.05) and if they lived with someone already vaccinated against COVID-19 (+6.4 percentage points, p<0.05).

**Table 4:** Multivariable ordinary least squares regression models of COVID-19 vaccine uptake among participants concerned about vaccine safety (model 1) and participants who do not believe the vaccine is effective (model 2)

	Model 1	Model 2
	Subgroup: believes vaccine is unsafe β (95% CI)	Subgroup: believes vaccine is not effective β (95% CI)
Perceived personal COVID-19 risk	0.129** (0.017–0.240)	0.169** (0.031–0.306)
Believes COVID-19 vaccine is effective	0.087** (0.021–0.154)	
Believes COVID-19 vaccine is unsafe		−0.049 (−0.124 to 0.025)
Perceived vaccination coverage: ‘Some’ people (Ref: Hardly any/Don’t know)	−0.016 (−0.078 to 0.046)	−0.043 (−0.102 to 0.017)
Perceived vaccination coverage: ‘Most’ people (Ref: Hardly any/Don’t know)	0.020 (−0.051 to 0.092)	0.035 (−0.037 to 0.108)
Lives with someone vaccinated	0.064** (0.010–0.118)	0.042 (−0.012 to 0.095)
Access barriers (scale 0–4)	0.028* (−0.002 to 0.058)	0.044** (0.009–0.079)
Control variables included	Yes	Yes
Observations	1707	1707
R^2^	0.363	0.365

***P<0.01, **p<0.05, *p<0.1.

Among participants who did not believe that the vaccine is effective, vaccine uptake was more likely among participants who thought they may get sick with COVID-19 within 12 months (+16.9 percentage points, p<0.05), and for those who reported greater access-related barriers (+4.4 percentage points per scale point, p<0.05). Results were substantively similar in models excluding the control variables (online supplemental table S6) and in logistic models (online supplemental table S7).

### Main reasons for getting vaccinated: Survey 2 qualitative responses

The most common reason that respondents gave for getting vaccinated was to protect themselves from COVID-19, with far fewer saying to protect those around them (online supplemental table S8). Mandates had the greatest influence on decisions, with one in five vaccinated because of employment-related mandates, and with other mandates also playing a role. Individuals got vaccinated both to keep their jobs and because they were looking for employment. Other, less common factors included seeing or becoming aware of others getting vaccinated; becoming aware of, or seeing first-hand, the negative health impacts of COVID-19; and a health status change (which generally captured the belief that it was not safe to get vaccinated while pregnant/breast feeding, or when sick with another illness).

## Discussion

Understanding factors that influence COVID-19 vaccine uptake is important both for continued efforts to increase coverage of COVID-19 vaccines and boosters, and for understanding how to prepare for future vaccine programmes. Using a longitudinal cohort in South Africa, this study demonstrated that stated vaccination intentions played a significant role in future self-reported vaccine uptake. This, together with the positive relationship found between vaccine uptake and attitudes, beliefs and subjective norms, is consistent with the mechanism of behaviour change posited by the theory of planned behaviour (TPB). Also aligned with the TPB, results indicate that the effect of attitudes, beliefs and subjective norms on vaccine uptake was mediated by vaccine intentions.

Three attitudes/beliefs central to behaviour change theories had a strong influence on stated intentions: people were more likely to want to get vaccinated if they perceived COVID-19 to present a personal health risk, if they believed the vaccine works and if they believed the vaccine is safe. The importance of these beliefs is underscored by the direct and large effect they also had on COVID-19 vaccine uptake. Furthermore, self-report reasons for why participants got vaccinated included to protect themselves (a predominant factor) and learning about the negative health impacts of COVID-19. While theoretically intuitive, these factors also likely played a significant role in impeding COVID-19 vaccine demand in South Africa, given how dynamic these beliefs were. Within 9 months of the start of the COVID-19 vaccine programme, fewer than half of our study participants (of unvaccinated individuals) believed that the vaccine works, two-thirds believed that the vaccine is unsafe and few perceived themselves at risk from COVID-19.

Given the proportion of respondents who believed the vaccine is unsafe and ineffective, and the tendency for these groups to be more hesitant towards vaccination, we explored factors that could potentially encourage COVID-19 vaccine uptake despite holding such beliefs. Among those who believed that the vaccine is unsafe, the perception that COVID-19 could make them sick and the belief that vaccine prevents getting and/or dying from COVID-19 encouraged vaccine uptake. Findings suggest that for some the perceived risks from getting vaccinated can be outweighed by the personal health benefits from vaccination. Among those not believing that the vaccine works, perceived personal risk from COVID-19 was the only predictor of vaccine uptake. This result may indicate that some individuals who do not believe the vaccine works (a measure which may capture individuals with varying strengths of this belief) will get vaccinated anyway in case there are health benefits from the vaccine.

Social norms were found to have a positive association with vaccine intentions. Participants who thought that most people in their area are vaccinated were more likely to want to get vaccinated, indicating the importance of descriptive norms (ie, what others are doing) in the formation of intentions. In addition, vaccine intentions were greater among participants who lived in a household with someone else who was vaccinated. First-hand contact with someone vaccinated may have influenced intentions through different mechanisms, and the influence of this person(s) may have been particularly important given they were likely part of the participant’s ingroup—that is, the influence someone has on you is greater if they are more like you.^42^ Someone in the household getting vaccinated could signal that it is something that ought to be done (injunctive norm), provide a cue to action, reduce frictions to getting vaccinated (eg, provide information on how to get vaccinated) and provide social proof that the vaccine is safe. The potential importance of seeing someone get vaccinated and remain healthy as social proof that the vaccine is safe is highlighted by both the finding that this factor influences vaccine uptake among participants who believed that the vaccine is unsafe, and by the finding that this was not a significant predictor of vaccine uptake in the full sample (ie, social proof was less important for people who thought the vaccine was safe). These quantitative findings are consistent with one theme that emerged as a reason given in open-ended responses for getting vaccinated: seeing or becoming aware of other people being vaccinated. While not all social proof interventions have proved effective,^43^ others provide positive evidence of the potential of this approach.^44 45^

Counter to expectations, our measure of perceived behavioural control had the opposite effect on vaccine intentions among the full sample, and on vaccine uptake among the subsample who did not believe the vaccine works. We postulate that this is a function of the survey items we used to proxy ‘perceived behavioural control’. Perceived behavioural control relates to a person’s belief that the target behaviour is within her/his control—it is conceptually similar to self-efficacy. It is typically measured with survey questions that assess the ease or difficulty of a behaviour, or an individual’s confidence in being able to enact a behaviour.^46^ Instead of measuring perceived behavioural control, our measure captures actual barriers to vaccination faced by study participants—what actually got in their way (ie, no time, living too far from a vaccination site, etc). The positive association between access barriers and the desire to get vaccinated helps explain why a significant proportion of the Survey 1 sample wanted to get vaccinated as soon as possible but had not yet done so several months after a vaccine was available to them. In line with this, two potential explanations for the positive association between access barriers in Survey 1 and subsequent vaccine uptake are the long summer holiday in South Africa that fell between Survey 1 and Survey 2, which may have enabled some participants to overcome a lack of time or distance to the clinic as barriers; and efforts to bring vaccination sites closer to people to reduce access barriers.^47^

Study findings have several implications. It is important for interventions to make salient the risk posed by a disease (without inducing unnecessary fear), as perceived personal risk may encourage vaccination, even among those who do not think the vaccine is effective. If the risks are relevant, then individuals may perceive it to be worth accepting what is being offered and recommended by health services. It is also important that vaccine programmes, from inception, are coupled with interventions that increase confidence and make salient the efficacy and safety of the vaccine. This is especially important in contexts where a vaccine was developed rapidly, which resulted in hesitancy towards the vaccine,^48^ and where scientific evidence and corresponding communication changed rapidly, as this undermined the credibility of information about COVID-19 and the vaccine(s).^48 49^ The roll-out of the COVID-19 vaccine happened at unprecedented speed globally. Consequently, given the vast numbers vaccinated, there were certainly many cases in which a COVID-19 vaccination coincided with a negative health event (eg, other disease, heart attack, etc), providing what would have been interpreted by some as proof that the vaccine is unsafe. It is therefore important to monitor and counter such beliefs frequently throughout a vaccine programme.

Our findings highlight the potential of social proof interventions as one way to overcome concerns about vaccine safety as a barrier to vaccination. As with other health-seeking behaviours, the success of promoting vaccination among one member of a household could have spillover effects in encouraging others to get vaccinated. This highlights the importance of helping individuals who want to get vaccinated to do so, by reducing frictions such as lacking time or living too far from a vaccination site. In addition, interventions to create awareness of, and make salient, vaccination as the social norm could directly increase intentions to get vaccinated and thereby vaccine coverage. A key lesson learnt that is reflected in our results as well as those of other teams studying COVID-19 vaccine demand is the importance of behavioural factors in both motivating vaccination and translating that motivation into action. Several resources have been developed based on lessons from COVID-19 to provide best practices for clear, engaging and actionable communication and interventions for different stages of a crisis.^50–53^ Future pandemic response efforts and vaccination campaigns should incorporate a behavioural science perspective into all stages of vaccination promotion and service delivery.^54 55^

Study findings should be interpreted in conjunction with its limitations. COVID-19 vaccine uptake, along with all measures of attitudes, beliefs and perceived personal control, was self-reported, and may have error due to self-reporting bias (although the alignment of the study findings with the TPB gives confidence in the data overall). As is typical with telephone surveys, attrition between Survey 1 and Survey 2 was large and may have resulted in selection bias, especially if attrition was correlated with vaccine uptake. While the sample frame (credit bureau database) included individuals across most of the socioeconomic spectrum in South Africa, it is likely the individuals at the extreme ends (those at the top who have never needed credit, and those at the bottom who have never applied for credit) were missing from the sample frame. This would also have introduced some selection bias.

In conclusion, our study underscores the importance of intentions in affecting behaviour and points to the importance of interventions that aim to promote beliefs that a disease poses personal risk and that a vaccine is safe and effective. Social norms or social proof-based interventions hold promise. With new COVID-19 variants that are more infectious than previous variants, and with declining herd immunity and immunity from outdated vaccines, learnings about vaccine uptake will be important both in the near future for COVID-19 vaccination and for demand-creation efforts for future vaccination campaigns.

## Data Availability

Data are available in a public, open access repository. Our CVACS data are publicly available at DataFirst: https://www.datafirst.uct.ac.za/dataportal/index.php/catalog/CVACS/?page=1&sort_by=title&sort_order=asc&ps=15&repo=CVACS

## References

[1] World health Organisation. WHO Coronavirus (COVID-19) dashboard. 2023. Available: https://covid19.who.int/ [Accessed 23 2023].

[2] Msemburi W, Karlinsky A, Knutson V, et al. The WHO estimates of excess mortality associated with the COVID-19 pandemic. Nature 2023;613:130–7. 10.1038/s41586-022-05522-2 36517599 PMC9812776

[3] Watson OJ, Barnsley G, Toor J, et al. Global impact of the first year of COVID-19 vaccination: a mathematical Modelling study. Lancet Infect Dis 2022;22:1293–302. 10.1016/S1473-3099(22)00320-6 35753318 PMC9225255

[4] Department of Health Republic of South Africa. Latest vaccine Statistics: overview of administered vaccines in the last 7 days. COVID-19 online resource and news portal. 2023. Available: https://sacoronavirus.co.za/latest-vaccine-statistics/

[5] Myburgh N, Mulaudzi M, Tshabalala G, et al. A qualitative study exploring Motivators and barriers to COVID-19 vaccine uptake among adults in South Africa and Zimbabwe. Vaccines (Basel) 2023;11:729. 10.3390/vaccines11040729 37112641 PMC10145404

[6] Solidarity Fund. Solidarity fund vaccine demand creation closing out campaign report. 2022. Available: https://solidarityfund.co.za/media/2022/03/SF_Vaccine_Demand_report_20032022.pdf [Accessed 27 2023].

[7] The COVID-19 pandemic in 2023: far from over. Lancet 2023;401:79. 10.1016/S0140-6736(23)00050-8 36641201

[8] Griffith J, Marani H, Monkman H. COVID-19 vaccine hesitancy in Canada: content analysis of Tweets using the theoretical domains framework. J Med Internet Res 2021;23:e26874. 10.2196/26874 33769946 PMC8045776

[9] Dror AA, Eisenbach N, Taiber S, et al. Vaccine hesitancy: the next challenge in the fight against COVID-19. Eur J Epidemiol 2020;35:775–9. 10.1007/s10654-020-00671-y 32785815 PMC8851308

[10] Brown P, Waite F, Larkin M, et al. It seems impossible that it’s been made so quickly”: a qualitative investigation of concerns about the speed of COVID-19 vaccine development and how these may be overcome. Hum Vaccin Immunother 2022;18:2004808. 10.1080/21645515.2021.2004808 35172678 PMC8928812

[11] Kricorian K, Civen R, Equils O. COVID-19 vaccine hesitancy: misinformation and perceptions of vaccine safety. Hum Vaccin Immunother 2022;18:1950504. 10.1080/21645515.2021.1950504 34325612 PMC8920251

[12] George G, Nota PB, Strauss M, et al. Understanding COVID-19 vaccine hesitancy among Healthcare workers in South Africa. Vaccines (Basel) 2023;11:414. 10.3390/vaccines11020414 36851290 PMC9966714

[13] Ackah BBB, Woo M, Stallwood L, et al. COVID-19 vaccine hesitancy in Africa: a Scoping review. Glob Health Res Policy 2022;7:1.:20. 10.1186/s41256-022-00255-1 35850783 PMC9294808

[14] Wiysonge CS, Alobwede SM, de Marie C Katoto P, et al. COVID-19 vaccine acceptance and hesitancy among healthcare workers in South Africa. Expert Review of Vaccines 2022;21:549–59. 10.1080/14760584.2022.2023355 34990311

[15] Trabucco Aurilio M, Mennini FS, Ferrari C, et al. Main predictors of COVID-19 vaccination uptake among Italian Healthcare workers in relation to variable degrees of hesitancy: result from a cross-sectional online survey. Trop Med Infect Dis 2022;7:419. 10.3390/tropicalmed7120419 36548674 PMC9780995

[16] Yan E, Lai DWL, Ng HKL, et al. Predictors of COVID-19 actual vaccine uptake in Hong Kong: A longitudinal population-based survey. SSM Popul Health 2022;18:101130. 10.1016/j.ssmph.2022.101130 35620485 PMC9119715

[17] Faries MD. “Why we don’t “just do it”: understanding the intention-behavior gap in lifestyle medicine”. Am J Lifestyle Med 2016;10:322–9. 10.1177/1559827616638017 30202289 PMC6125069

[18] Wand H, Vujovich-Dunn C, Moodley J, et al. Population-level impact of beliefs and attitudes on vaccine decision-making in South Africa: results from the COVID-19 vaccine survey (2021/2022). Public Health 2023;216:58–65. 10.1016/j.puhe.2023.01.007 36801593 PMC9829597

[19] Ajzen I. The theory of planned behavior. Organizational Behavior and Human Decision Processes 1991;50:179–211. 10.1016/0749-5978(91)90020-T

[20] Ajzen I. From intentions to actions: a theory of planned behavior. Action Control 1985:11–39. 10.1007/978-3-642-69746-3

[21] Kan MPH, Fabrigar LR. Theory of planned behavior. Encyclopedia of Personality and Individual Differences 2017:1–8. 10.1007/978-3-319-28099-8

[22] Takuva S, Takalani A, Seocharan I, et al. Safety evaluation of the single-dose Ad26. Cov2.S vaccine among Healthcare workers in the Sisonke study in South Africa: A phase 3B implementation trial. PLoS Med 2022;19:e1004024. 10.1371/journal.pmed.1004024 35727802 PMC9212139

[23] Worldometer. Worldometer COVID-19 data: daily new deaths in South Africa. Worldometer. Available: https://www.worldometers.info/coronavirus/country/south-africa/ [Accessed 6 2023].

[24] Southern Africa Labour and Development Research Unit. COVID-19 vaccine survey (CVACS) 2021, survey 1 [Dataset]. version 1.1.0; 2022.

[25] Southern Africa Labour and Development Research Unit. COVID-19 vaccine survey (CVACS) 2022, survey 2 [Dataset]. version 1; 2022.

[26] Brophy T, Ingle K, Maughan-Brown B, et al. COVID-19 vaccine survey (CVACS)2021: panel user manual. Cape Town; 2022.

[27] Javadi M, Kadkhodaee M, Yaghoubi M, et al. Applying theory of planned behavior in predicting of patient safety behaviors of nurses. Mater Sociomed 2013;25:52–5. 10.5455/msm.2013.25.52-55 23687461 PMC3655788

[28] Khayyam M, Chuanmin S, Salim MA, et al. COVID-19 vaccination behavior among frontline Healthcare workers in Pakistan: the theory of planned behavior, perceived susceptibility, and anticipated regret. Front Psychol 2022;13:808338. 10.3389/fpsyg.2022.808338 35496249 PMC9050246

[29] Wolff K. COVID-19 vaccination intentions: the theory of planned behavior, optimistic bias, and anticipated regret. Front Psychol 2021;12:2404. 10.3389/fpsyg.2021.648289 34220620 PMC8241938

[30] Pourmand G, Doshmangir L, Ahmadi A, et al. An application of the theory of planned behavior to self-care in patients with hypertension. BMC Public Health 2020;20:1–8. 10.1186/s12889-020-09385-y 32847501 PMC7448508

[31] Karlsson LC, Soveri A, Lewandowsky S, et al. Fearing the disease or the vaccine: the case of COVID-19. Pers Individ Dif 2021;172:110590. 10.1016/j.paid.2020.110590 33518869 PMC7832025

[32] Burger R, Köhler T, Golos AM, et al. Longitudinal changes in COVID-19 vaccination intent among South African adults: evidence from the NIDS-CRAM panel survey, February to may 2021. BMC Public Health 2022;22:1–10. 10.1186/s12889-022-12826-5 35236319 PMC8889513

[33] Kahn K, Pettifor A, Mataboge P, et al. COVID-19 vaccine hesitancy in rural South Africa: deepening understanding to increase uptake and access. J Glob Health 2022;12:1–7. 10.7189/jogh.12.05013 35567586 PMC9107307

[34] Kafadar AH, Tekeli GG, Jones KA, et al. Determinants for COVID-19 vaccine hesitancy in the general population: a systematic review of reviews. Z Gesundh Wiss 2022:1–17. 10.1007/s10389-022-01753-9 36160668 PMC9483252

[35] StataCorp. Stata statistical software: release 17. College Station; 2021.

[36] Bengtsson M. How to plan and perform a qualitative study using content analysis. NursingPlus Open 2016;2:8–14. 10.1016/j.npls.2016.01.001

[37] Morgan DL. Qualitative content analysis: a guide to paths not taken. Qual Health Res 1993;3:112–21. 10.1177/104973239300300107 8457790

[38] Raskind IG, Shelton RC, Comeau DL, et al. A review of qualitative data analysis practices in health education and health behavior research. Health Educ Behav 2019;46:32–9. 10.1177/1090198118795019 30227078 PMC6386595

[39] Sam-Agudu NA, Abimbola S. Using scientific authorship criteria as a tool for equitable inclusion in global health research. BMJ Glob Health 2021;6:e007632. 10.1136/bmjgh-2021-007632 34649868 PMC8506888

[40] Morton B, Vercueil A, Masekela R, et al. Consensus statement on measures to promote equitable authorship in the publication of research from international partnerships. Anaesthesia 2022;77:264–76. 10.1111/anae.15597 34647323 PMC9293237

[41] Draaijer M, Lalla-Edward ST, Venter WDF, et al. Phone calls to retain research participants and determinants of Reachability in an African setting: observational study. JMIR Form Res 2020;4:e19138. 10.2196/19138 32996891 PMC7557447

[42] Goldstein NJ, Cialdini RB, Griskevicius V. A room with a viewpoint: using social norms to motivate environmental conservation in hotels. J Consum Res 2008;35:472–82. 10.1086/586910/2/35-3-472-FG4.JPEG

[43] Rabb N, Swindal M, Glick D, et al. Evidence from a statewide vaccination RCT shows the limits of Nudges. Nature 2022;604:E1–7. 10.1038/s41586-022-04526-2 35388200

[44] Sasaki S, Saito T, Ohtake F. Nudges for COVID-19 voluntary vaccination: how to explain peer information? Soc Sci Med 2022;292. 10.1016/j.socscimed.2021.114561 34823128 PMC8577869

[45] Moehring A, Collis A, Garimella K, et al. Surfacing norms to increase vaccine acceptance. MIT Initiative On The Digital Economy 2021;2. 10.2139/ssrn.3782082

[46] Wallston K. Control beliefs: health perspectives. International Encyclopedia of the Social & Behavioral Sciences 2001:2724–6. 10.1016/B0-08-043076-7/03799-2

[47] Casas MG. How Vaxi TAXI is Repurposing public spaces and Partnering with Western Cape communities to bridge the vaccination gap. daily maverick. 2021. Available: https://www.dailymaverick.co.za/article/2021-12-21-how-vaxi-taxi-is-repurposing-public-spaces-and-partnering-with-western-cape-communities-to-bridge-the-vaccination-gap/

[48] Machingaidze S, Wiysonge CS. Understanding COVID-19 vaccine hesitancy. Nat Med 2021;27:1338–9. 10.1038/s41591-021-01459-7 34272500

[49] Lockyer B, Islam S, Rahman A, et al. Understanding COVID-19 misinformation and vaccine hesitancy in context: findings from a qualitative study involving citizens in Bradford, UK. Health Expect 2021;24:1158–67. 10.1111/hex.13240 33942948 PMC8239544

[50] Asian Development Bank, McCann Global Health. COVID-19 risk communications promising practices Playbook. Manila, Philippines: 2021, 10.22617/TCS200350-2

[51] Centers for Disease Control and Prevention (CDC). COVID-19 Vaccinationprogram interim operational guidance jurisdiction operations. 2020. Available: https://www.cdc.gov/vaccines/covid-19/covid19-vaccination-guidance.html#guidance-jurisdictions [Accessed 8 Jun 2023].

[52] Breakthrough ACTION, ideas42. BEHAVORIAL INSIGHTS behavioral INSIGHTS DESIGN MENU. 2022. Available: https://breakthroughactionandresearch.org/closing-covid-19-vaccine-gap-among-health-workers-in-lmic/ [Accessed 8 Jun 2023].

[53] Center for Health Incentives and Behavioral Economics (CHIBE). Science-informed guide and tip sheets support response to COVID-19 inLow-and-middle-income countries. 2020. Available: https://chibe.upenn.edu/chibeblog/behavioral-science-informed-guide-and-tip-sheets-support-response-to-covid-19-in-low-and-middle-income-countries/ [Accessed 8 Jun 2023].

[54] Dhama K, Sharun K, Tiwari R, et al. COVID-19 vaccine hesitancy - reasons and solutions to achieve a successful global vaccination campaign to tackle the ongoing pandemic. Hum Vaccin Immunother 2021;17:3495–9. 10.1080/21645515.2021.1926183 34191680 PMC8437517

[55] Batteux E, Mills F, Jones LF, et al. The effectiveness of interventions for increasing COVID-19 vaccine uptake: A systematic review. Vaccines (Basel) 2022;10. 10.3390/vaccines10030386 35335020 PMC8949230

