# Supplementary material for: Predictors of COVID-19 vaccine uptake among adults in South Africa: multimethod evidence from a population-based longitudinal study
Source: BMJ Glob Health. 2023 Aug 4;8(8):e012433. doi: 10.1136/bmjgh-2023-012433 (PMC10407380; doi:10.1136/bmjgh-2023-012433)
Supplement: Supplementary data [file bmjgh-2023-012433supp002.pdf]

**Appendix S1 – Reflexivity Statement**

**Article:** Predictors of COVID-19 vaccine uptake among adults in South Africa: Multi-method evidence from a population-based longitudinal study

**1. How does this study address local research and policy priorities?**

Our study sought to understand and address South Africa's pressing public health challenge of COVID-19 vaccine hesitancy. The findings and recommendations were aligned with the policy priorities of promoting vaccine uptake and managing the COVID-19 pandemic. The study also addressed local research priorities by generating new knowledge about vaccine acceptance and hesitancy among South Africans.

**2. How were local researchers involved in study design?**

Local researchers played key roles in the study design, contributing to the formulation of the survey questionnaire, and the selection of appropriate sampling techniques. In addition to the local research team, we consulted local NGOs, health journalists, and government officials during the design phase to ensure the relevance and inclusivity of the study. The CVACS Principal Investigators are South African and based at the University of Cape Town.

**3. How has funding been used to support the local research team?**

The funding received from the Bill and Melinda Gates Foundation was used to support the local research team by facilitating their participation in the study design, data collection, analysis, and dissemination of results. The funding was used to compensate the team for their time and expertise, provide necessary training, and purchase equipment and resources needed for the study.

**4. How are research staff who conducted data collection acknowledged?**

The research staff who conducted the data collection were acknowledged publicly in policy briefings, webinars, and data dissemination activities. Their efforts were also recognized in the final study report and associated publications.



Maughan-Brown B, *et al. BMJ Glob Health* 2023; 8:e012433. doi: 10.1136/bmjgh-2023-012433

**5. Do all members of the research partnership have access to study data?**

Yes, all members of the research partnership have access to the study data. Our data is publicly available at DataFirst, signifying our commitment to transparency and openness.

**6. How was data used to develop analytical skills within the partnership?**

The data analysis process was conducted as a collaborative exercise, with local and international researchers working together. This collaborative approach provided opportunities for capacity building and skills development among the local research team, enhancing their analytical skills.

**7. How have research partners collaborated in interpreting study data?**

Research partners worked collaboratively in interpreting the study data. Regular meetings and workshops were held during the data analysis phase to discuss the results, draw conclusions, and develop policy recommendations. These collaborative efforts ensured that multiple perspectives and interpretations were considered in our analysis.

**8. How were research partners supported to develop writing skills?**

Junior researchers, including early career researchers and PhD candidates, were given opportunities to contribute to the writing of the final report and other publications. They were supported by senior researchers in their writing process, enhancing their academic writing skills.

**9. How will research products be shared to address local needs?**

Our study findings will be disseminated widely through policy briefings, public webinars, and academic publications. The findings will also be shared with stakeholders, including local NGOs, health journalists, and government officials, to inform vaccine demand creation campaigns and other interventions aimed at addressing vaccine hesitancy. Our CVACS data is publicly available at DataFirst, signifying our commitment to transparency and openness.

**10. How is the leadership, contribution and ownership of this work by LMIC researchers recognised within the authorship?**

Our research involved meaningful contributions from researchers based in South Africa, a LMIC. Their leadership roles in the study are recognized in the authorship of the publications arising from this research. They were given prominent positions in the authorship list, reflecting their substantial contributions to the study.

11. **How have early career researchers across the partnership been included within the authorship team?**

We consciously included early career researchers in the authorship team. They were involved in all aspects of the research process, from design to data analysis, and were given the opportunity to contribute to writing the study report and other publications.

**12. How has gender balance been addressed within the authorship?**

The authorship team reflected a balance in terms of gender (two women and two men). Both men and women researchers, including the principal investigator and key members of the data collection and processing team, were included in the authorship list.

**13. How has the project contributed to training of LMIC researchers?**

The project provided opportunities for capacity building among local researchers. They were involved in all stages of the research process, from study design to data analysis, which enhanced their research skills. Furthermore, early career researchers and PhD candidates were given the opportunity to contribute to writing the study report and other publications, developing their academic writing skills.

**14. How has the project contributed to improvements in local infrastructure?**

While the project did not directly invest in physical infrastructure, it contributed to strengthening local research capacity and infrastructure. The experience and skills gained by the local research team, the establishment of collaborative networks, and the generation of new knowledge about vaccine hesitancy among South Africans all contribute to enhancing the local research ecosystem.



Maughan-Brown B, *et al. BMJ Glob Health* 2023; 8:e012433. doi: 10.1136/bmjgh-2023-012433

**15. What safeguarding procedures were used to protect local study participants and researchers?**

We implemented robust safeguarding procedures to ensure the privacy and confidentiality of study participants. This included obtaining informed consent from all participants and adhering to ethical guidelines for data collection and storage. For researchers, we ensured a safe and respectful working environment, and provided necessary support and resources for their roles in the study.



Maughan-Brown B, *et al. BMJ Glob Health* 2023; 8:e012433. doi: 10.1136/bmjgh-2023-012433
