# Supplementary material for: Predictors of COVID-19 vaccine uptake among adults in South Africa: multimethod evidence from a population-based longitudinal study
Source: BMJ Glob Health. 2023 Aug 4;8(8):e012433. doi: 10.1136/bmjgh-2023-012433 (PMC10407380; doi:10.1136/bmjgh-2023-012433)
Supplement: Supplementary data [file bmjgh-2023-012433supp001.pdf]

Supplemental Tables

Article: Predictors of COVID-19 vaccine uptake among adults in South Africa: Multi-method evidence from a population-based longitudinal study

Table S1. Multivariable ordinary least squares regression models of COVID-19 vaccine intention (dependent variable: plans to get vaccinated as soon as possible, Survey 1) without control variables.

	**β** (95% CI)	**β** (95% CI)
Perceived personal COVID-19 risk	0.087*	0.087*
	[-0.002 - 0.176]	[-0.001 - 0.176]
Believes COVID-19 vaccine is effective	0.333***	0.331***
	[0.268 - 0.397]	[0.267 - 0.396]
Believes COVID-19 vaccine is unsafe	-0.194***	-0.192***
	[-0.256 - -0.132]	[-0.254 - -0.130]
Perceived vaccination coverage: "Some" people [Ref: hardly any/Don't know]	-0.007	-0.008
	[-0.065 - 0.051]	[-0.065 - 0.050]
Perceived vaccination coverage: "Most" people [Ref: hardly any/Don't know]	0.078**	0.079**
	[0.009 - 0.147]	[0.010 - 0.147]
Lives with someone vaccinated	0.067**	0.065**
	[0.015 - 0.119]	[0.014 - 0.117]
Access barriers (Scale 0-4)	0.086***	
	[0.059 - 0.113]	
Reason not vaccinated: Does not have time		0.111***
		[0.046 - 0.176]
Reason not vaccinated: Do not know where to go		0.093**
		[0.010 - 0.177]
Reason not vaccinated: Site too far away		0.058
		[-0.033 - 0.149]
Reason not vaccinated: Transport too costly		0.082**
		[0.009 - 0.155]
Control variables included	No	No
Observations	1,707	1,707
R-squared	0.363	0.365

*** p<0.01, ** p<0.05, * p<0.1

Table S2. Multivariable logistic regression models of COVID-19 vaccine intention (dependent variable: plans to get vaccinated as soon as possible, Survey 1).

	OR (95% CI)	OR (95% CI)
Perceived personal COVID-19 risk	1.825**	1.805**
	[1.058 - 3.149]	[1.048 - 3.109]
Believes COVID-19 vaccine is effective	5.998***	5.905***
	[4.191 - 8.582]	[4.119 - 8.467]
Believes COVID-19 vaccine is unsafe	0.293***	0.299***
	[0.202 - 0.424]	[0.206 - 0.433]
Perceived vaccination coverage: "Some" people [Ref: hardly any/Don't know]	0.984	0.972
	[0.606 - 1.597]	[0.602 - 1.570]
Perceived vaccination coverage: "Most" people [Ref: hardly any/Don't know]	1.640**	1.635**
	[1.022 - 2.633]	[1.020 - 2.619]
Lives with someone vaccinated	1.545**	1.547**
	[1.077 - 2.217]	[1.077 - 2.221]
Access barriers (Scale 0-4)	1.669***	
	[1.399 - 1.992]	
Reason not vaccinated: Does not have time		2.193***
		[1.480 - 3.250]
Reason not vaccinated: Do not know where to go		1.436
		[0.855 - 2.414]
Reason not vaccinated: Site too far away		1.428
		[0.842 - 2.424]
Reason not vaccinated: Transport too costly		1.634*
		[0.988 - 2.702]
Control variables included	Yes	Yes
Observations	1,707	1,707

*** p<0.01, ** p<0.05, * p<0.1

Table S3. Multivariable ordinary least squares regression models of COVID-19 vaccine intention (dependent variable: plans to get vaccinated as soon as possible, Survey 1) among the full CVACS Survey 1 sample.

	**β** (95% CI)	**β** (95% CI)
Perceived personal COVID-19 risk	0.063*	0.059*
	[-0.003 - 0.129]	[-0.006 - 0.124]
Believes COVID-19 vaccine is effective	0.314***	0.304***
	[0.266 - 0.362]	[0.256 - 0.351]
Believes COVID-19 vaccine is unsafe	-0.194***	-0.184***
	[-0.241 - -0.147]	[-0.231 - -0.137]
Perceived vaccination coverage: "Some" people [Ref: hardly any/Don't know]	0.005	-0.002
	[-0.038 - 0.049]	[-0.045 - 0.040]
Perceived vaccination coverage: "Most" people [Ref: hardly any/Don't know]	0.069***	0.062**
	[0.018 - 0.119]	[0.011 - 0.112]
Lives with someone vaccinated	0.072***	0.070***
	[0.033 - 0.112]	[0.031 - 0.110]
Access barriers (Scale 0-4)	0.079***	
	[0.059 - 0.100]	
Reason not vaccinated: Does not have time		0.178***
		[0.129 - 0.227]
Reason not vaccinated: Do not know where to go		-0.020
		[-0.081 - 0.042]
Reason not vaccinated: Site too far away		0.113***
		[0.044 - 0.182]
Reason not vaccinated: Transport too costly		0.014
		[-0.048 - 0.075]
Control variables included	Yes	Yes
Observations	3,157	3,157
R-squared	0.321	0.333

*** p<0.01, ** p<0.05, * p<0.1

Table S4. Multivariable ordinary least squares regression models of COVID-19 vaccine uptake (dependent variable: vaccine status in Survey 2).

	**β** (95% CI)	**β** (95% CI)	**β** (95% CI)	**β** (95% CI)	**β** (95% CI)
Vaccine intention: As soon as possible [Ref: everyone else]	0.272*** [0.211 - 0.332]			0.208*** [0.130 - 0.285]	
Vaccine intention: Definitely not [Ref: "wait and see"]		-0.093*** [-0.145 - -0.042]			-0.071*** [-0.124 - -0.019]
Vaccine intention: Only if Required [Ref: "wait and see"]		-0.032 [-0.093 - 0.029]			-0.017 [-0.077 - 0.043]
Vaccine intention: As soon as possible [Ref: "wait and see"]		0.232*** [0.160 - 0.304]			0.189*** [0.106 - 0.271]
Vaccine intention: Don't know [Ref: "wait and see"]		0.005 [-0.115 - 0.124]			0.014 [-0.106 - 0.134]
Perceived personal COVID-19 risk			0.060 [-0.035 - 0.155]	0.042 [-0.047 - 0.130]	0.043 [-0.045 - 0.131]
Believes COVID-19 vaccine is effective			0.133*** [0.074 - 0.192]	0.065** [0.003 - 0.126]	0.054* [-0.009 - 0.118]
Believes COVID-19 vaccine is unsafe			-0.097*** [-0.158 - -0.035]	-0.056* [-0.120 - 0.007]	-0.055* [-0.118 - 0.008]
Perceived vaccination coverage: "Some" people [Ref: hardly any/Don't know]			0.013 [-0.045 - 0.071]	0.014 [-0.041 - 0.069]	0.013 [-0.042 - 0.068]
Perceived vaccination coverage: "Most" people [Ref: hardly any/Don't know]			0.037 [-0.027 - 0.102]	0.019 [-0.043 - 0.082]	0.016 [-0.046 - 0.078]
Lives with someone vaccinated			0.020 [-0.031 - 0.071]	0.007 [-0.041 - 0.056]	0.004 [-0.045 - 0.052]

Access barriers (Scale 0-4)			0.023* [-0.004 - 0.050]	0.006 [-0.022 - 0.033]	0.003 [-0.025 - 0.031]
Control variables included	No	No	No	No	No
Observations	1,767	1,767	1,757	1,755	1,755
R-squared	0.103	0.110	0.077	0.117	0.122

*** p<0.01, ** p<0.05, * p<0.1

Table S5. Multivariable logistic regression models of COVID-19 vaccine uptake (dependent variable: vaccine status in Survey 2).

	aOR (95% CI)	aOR (95% CI)	aOR (95% CI)	aOR (95% CI)	aOR (95% CI)
Vaccine intention: As soon as possible [Ref: everyone else]	5.254***			3.503***	
	[3.658 - 7.546]			[2.187 - 5.611]	
Vaccine intention: Definitely not [Ref: "wait and see"]		0.361***			0.431**
		[0.187 - 0.700]			[0.219 - 0.849]
Vaccine intention: Only if Required [Ref: "wait and see"]		0.826			0.937
		[0.474 - 1.438]			[0.540 - 1.629]
Vaccine intention: As soon as possible [Ref: "wait and see"]		3.986***			3.075***
		[2.549 - 6.233]			[1.846 - 5.120]
Vaccine intention: Don't know [Ref: "wait and see"]		1.184			1.281
		[0.453 - 3.098]			[0.491 - 3.342]
Perceived personal COVID-19 risk			1.679**	1.514	1.516
			[1.000 - 2.820]	[0.916 - 2.501]	[0.920 - 2.496]
Believes COVID-19 vaccine is effective			2.287***	1.505*	1.389
			[1.535 - 3.409]	[0.965 - 2.349]	[0.891 - 2.167]
Believes COVID-19 vaccine is unsafe			0.575***	0.725	0.734
			[0.400 - 0.827]	[0.492 - 1.069]	[0.500 - 1.077]
Perceived vaccination coverage: "Some" people [Ref: hardly any/Don't know]			1.103	1.116	1.116
			[0.687 - 1.771]	[0.695 - 1.790]	[0.697 - 1.787]
Perceived vaccination coverage: "Most" people [Ref: hardly any/Don't know]			1.427	1.287	1.268
			[0.895 - 2.275]	[0.803 - 2.063]	[0.793 - 2.026]
Lives with someone vaccinated			1.107	1.009	0.996
			[0.769 - 1.595]	[0.702 - 1.450]	[0.694 - 1.430]
Access barriers (Scale 0-4)			1.160*	1.055	1.035
			[0.992 - 1.357]	[0.889 - 1.252]	[0.873 - 1.228]
Control variables included	Yes	Yes	Yes	Yes	Yes
Observations	1,767	1,767	1,757	1,755	1,755

*** p<0.01, ** p<0.05, * p<0.1

Table S6. Multivariable ordinary least squares regression models of COVID-19 vaccine uptake among participants concerned about vaccine uptake (Model 1) and participants who do not believe the vaccine is effective (Model 2)

	Subgroup: vaccine unsafe	Subgroup: vaccine not effective
	**β** (95% CI)	**β** (95% CI)
Perceived personal COVID-19 risk	0.123**	0.183**
	[0.011 - 0.235]	[0.040 - 0.327]
Believes COVID-19 vaccine is effective	0.100***	
	[0.035 - 0.166]	
Believes COVID-19 vaccine is unsafe		-0.068*
		[-0.143 - 0.006]
Perceived vaccination coverage: "Some" people [Ref: hardly any/Don't know]	-0.015	-0.043
	[-0.075 - 0.046]	[-0.102 - 0.017]
Perceived vaccination coverage: "Most" people [Ref: hardly any/Don't know]	0.012	0.007
	[-0.059 - 0.083]	[-0.066 - 0.081]
Lives with someone vaccinated	0.069**	0.036
	[0.015 - 0.122]	[-0.017 - 0.089]
Access barriers (Scale 0-4)	0.035**	0.046**
	[0.005 - 0.065]	[0.009 - 0.082]
Control variables included	No	No
Observations	1,707	1,707
R-squared	0.363	0.365

Table S7. Multivariable logistic regression models of COVID-19 vaccine uptake among participants concerned about vaccine uptake (Model 1) and participants who do not believe the vaccine is effective (Model 2)

	Subgroup: vaccine unsafe	Subgroup: vaccine not effective
	aOR (95% CI)	aOR (95% CI)
Perceived personal COVID-19 risk	2.433***	3.563***
	[1.272 - 4.651]	[1.683 - 7.545]
Believes COVID-19 vaccine is effective	1.844**	
	[1.110 - 3.063]	
Believes COVID-19 vaccine is unsafe		0.641
		[0.336 - 1.222]
Perceived vaccination coverage: "Some" people [Ref: hardly any/Don't know]	0.871	0.577
	[0.478 - 1.585]	[0.285 - 1.169]
Perceived vaccination coverage: "Most" people [Ref: hardly any/Don't know]	1.250	1.368
	[0.681 - 2.293]	[0.681 - 2.749]
Lives with someone vaccinated	1.687**	1.563
	[1.067 - 2.665]	[0.910 - 2.684]
Access barriers (Scale 0-4)	1.228**	1.507***
	[1.001 - 1.506]	[1.163 - 1.952]
Control variables included	Yes	Yes
Observations	1,130	958

Table S8. Reasons for getting vaccinated from content analysis of Survey 2 responses

Main reason?	%	What changed?	%
Protect myself from COVID	35,6	Mandates - employment related	20,8
Mandates - employment related	20,9	Protect myself from COVID	16,9
Mandates - all except employment	5,2	Mandates - all except employment	7,0
Protect those around me	5,2	Saw or aware of many or some other people vaccinated	6,3
Uncategorised	4,8	Health status change	5,2
Saw or aware of many or some other people vaccinated	4,3	Witnessed or aware of COVID sickness or death	5,2
Witnessed or aware of COVID sickness or death	3,7	Nothing changed or I just decided	5,1
Told or advised to get vaccinated	3,1	Uncategorised	4,9
Learned or saw vaccine is safe	2,7	Increased vaccine access or had more time	4,5
Nothing changed or I just decided	2,5	Protect those around me	3,8
